# Differential airway remodeling changes were observed in patients with asthma COPD overlap compared to patients with asthma and COPD alone

**DOI:** 10.1152/ajplung.00137.2022

**Published:** 2022-08-23

**Authors:** Surajit Dey, Wenying Lu, Heinrich C. Weber, Sally Young, Josie Larby, Collin Chia, Greg Haug, Samuel James Brake, Stephen Myers, Archana Vijay Gaikwad, Prem Bhattarai, Prabuddha S. Pathinayake, Peter A. B. Wark, Mathew Suji Eapen, Sukhwinder Singh Sohal

**Affiliations:** ^1^Respiratory Translational Research Group, Department of Laboratory Medicine, School of Health Sciences, College of Health and Medicine, University of Tasmania, Launceston, Tasmania, Australia; ^2^Department of Respiratory Medicine, Tasmanian Health Services (THS), North-West Hospital, Burnie, Tasmania, Australia; ^3^Lung Function Unit, North-West Regional Hospital, Burnie, Tasmania, Australia; ^4^Department of Respiratory Medicine, Launceston General Hospital, Launceston, Tasmania, Australia; ^5^Priority Research Centre for Healthy Lungs, Hunter Medical Research Institute, University of Newcastle, New Lambton Heights, New South Wales, Australia; ^6^Department of Respiratory and Sleep Medicine John Hunter Hospital, New Lambton Heights, New South Wales, Australia

**Keywords:** airway remodeling, asthma, asthma COPD overlap, COPD, smoking

## Abstract

Management of patients with asthma COPD overlap (ACO) is clinically challenging due to insufficient evidence of pathological changes in these patients. In this cross-sectional study, we evaluated airway remodeling in endobronchial biopsies from a total of 90 subjects, which included 12 ACO, 14 patients with asthma, 12 COPD exsmokers (ES), 11 current smokers (CS), 28 healthy controls (HC), and 13 normal lung function smokers (NLFS). Tissue was stained with Masson’s trichrome. Epithelium, goblet cells, reticular basement membrane (RBM), cellularity, lamina propria (LP), and smooth muscle (SM) changes were measured using Image-Pro Plus v7 software. Differential airway remodeling pattern was seen in patients with ACO. A limited change was noted in the ACO epithelium compared with other pathological groups. RBM was substantially thicker in patients with ACO than in HC (*P* < 0.0002) and tended to be thicker than in patients with asthma and NLFS. The total RBM cells were higher in ACO than in the HC (*P* < 0.0001), COPD-CS (*P* = 0.0559), -ES (*P* = 0.0345), and NLFS (*P* < 0.0002), but did not differ from patients with asthma. Goblet cells were higher in the ACO than in the HC (*P* = 0.0028) and COPD-ES (*P* = 0.0081). The total LP cells in ACO appeared to be higher than in HC, COPD-CS, and NLFS but appeared to be lower than in patients with asthma. Finally, SM area was significantly lower in the ACO than in patients with asthma (*P* = 0.001), COPD-CS (=0.0290), and NLFS (*P* = 0.0011). This first comprehensive study suggests that patients with ACO had distinguishable tissue remodeling that appeared to be more severe than patients with asthma and COPD. This study will help in informed decision-making for better patient management in clinical practice.

## Graphical abstract

**Figure:**
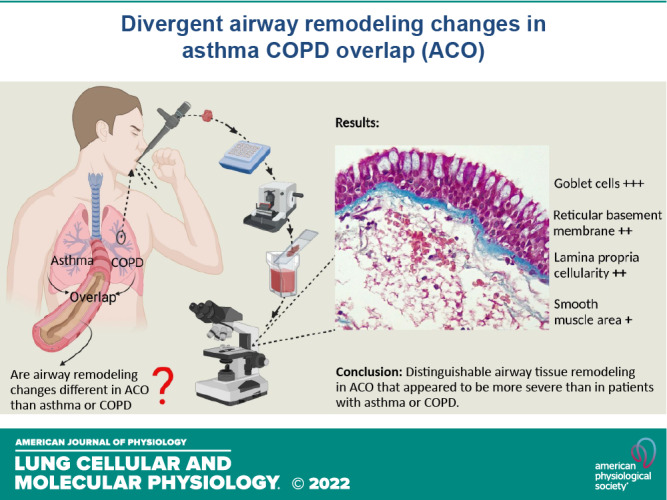

## INTRODUCTION

Asthma and chronic obstructive pulmonary disease (COPD) are heterogeneous human lung diseases, with distinctive inflammatory patterns and tissue structures afflicting different anatomical locations (1, 2). Asthma is associated with airway hyperresponsiveness and reversible airflow limitation, whereas persistent respiratory symptoms and airflow limitations characterize COPD. Major airway structural changes in patients with asthma include goblet and mucous gland hyperplasia, smooth muscle hypertrophy, angiogenesis, and epithelial alterations (3, 4). On the other hand, in COPD, some of the featured remodeling changes include squamous cell metaplasia, goblet cell hyperplasia, reticular basement membrane (RBM) fragmentation, epithelial-to-mesenchymal transition (EMT), altered vascularity, RBM, and lamina propria (LP) cellularity (4–13). Furthermore, small airway fibrosis and thickening and emphysema are variably observed in patients with COPD. Inflammation influences these changes in both asthma and COPD and can vary depending on their age and disease stage (1, 2). Asthma COPD overlap (ACO) is a descriptive clinical term to identify patients presenting with features of both asthma and COPD (1, 2, 14). Our understanding of ACO is at a very preliminary stage. However, it is quite likely that ACO is consistently misdiagnosed and suboptimally managed as “only” asthma or COPD. One major reason for this presumed lack of diagnosis is the absence of detailed studies of the airway pathological changes associated with ACO.

Several overviews (15, 16) on ACO have been published; however, a few direct tissue-based observational studies have made an in-depth assessment of airway remodeling in ACO. Previous analysis using computed tomography (CT) studies in patients with ACO indicated significant airway wall thickening in ACO compared with either asthma or COPD (17, 18). In addition, some endobronchial biopsy (EBB) studies suggested the thickening of RBM as the differentiating factor for ACO (17, 19). However, these studies were either qualitative or were limited to a few remodeling parameters; therefore, a two-dimensional quantitative assessment will be helpful to understand the various microstructural features of airway remodeling and its inflammatory consequences.

As asthma and COPD are both chronic lung diseases with distinguishable airway remodeling changes, it is expected that a differing phenotype could emerge when the pathologies overlap. We hypothesize that a distinct remodeling pattern exists in the airway of patients with ACO. Therefore, in this study, we conducted an in-depth histopathological-based morphometric study evaluating large airway biopsies, specifically focusing on the mucosal epithelium, reticular basement membrane, lamina propria, and the smooth muscle area in clinically well-characterized patients with ACO. In addition, we make critical comparisons within similar areas for patients with asthma, COPD current, and exsmokers, smokers with normal lung function, and healthy nonsmoking subjects.

## METHODS

### Collection of Endobronchial Biopsies and Demographics

A total of 90 large airway EBB samples (12 ACO, 14 with asthma, 23 COPD, 28 healthy, and 13 NLFS) were obtained from the Tasmanian Respiratory and Newcastle biobanks (the Hunter New England Human Research Ethics Committee Reference no: 05/08/10/3.09; Tasmanian Health and Medical Human Research Ethics Committee, ethics ID: H0013051). These EBB samples were collected from adult research volunteers, who provided written informed consent. Bronchoscopy procedures were performed by the respiratory physician as per the routine hospital protocol. Subjects were premedicated with topical lignocaine at a dose not exceeding 300 mg, 2.5–10 mg of midazolam IVI, and 50–100 µg of fentanyl IVI. Subjects were monitored by pulse oximetry and administered oxygen during the procedure. Three to eight biopsies were taken from each volunteer from the right lower lobe’s secondary carina of segmental and subsegmental bronchi. There were no complications from the procedures. Post biopsy, bronchial samples were fixed in 4% neutral-buffered formalin for 2 h before being processed into paraffin with a Leica ASP 200 tissue processor using graded alcohol and xylene.

Patients with asthma had a physician’s diagnosis of asthma with objective evidence of airflow variability or bronchial hyperactivity (1). COPD diagnoses criteria was based on Global Initiative for Chronic Obstructive Lung Disease (GOLD) guidelines (2). ACO was defined using a combination of COPD with asthma definition: evidence of respiratory symptoms, history of asthma, allergy, or atopy in combination with a postbronchodilator forced expiratory volume in 1 s (FEV_1_) < 80% of predicted value and FEV_1_/forced vital capacity (FVC) <70% plus an increase in postbronchodilator FEV_1_ or FVC ≥200 mL and ≥12% (1, 2, 14). All HC had no previous history of respiratory illness and had normal lung function assessed by spirometry. The NLFS had normal spirometry values (FEV_1_ >80% of predicted and FEV_1_/FVC >70%) and did not deviate from the expiratory descending arm flow-volume curve. Among the patients with ACO in our study, seven were classified as GOLD stage I and II COPD, one with GOLD stage III attributed to their lung function, and four patients were classified as severe asthma. Most patients with ACO were exsmokers with a smoking history of 22.5 pack·years. All patients with COPD were mild-moderate (GOLD stage I and II), of which 11 were current smokers (COPD-CS) with a smoking history of 46.3 pack·years and 12 were COPD exsmokers (COPD-ES) with a smoking history of 62 pack·years. NLFS smoked a minimum of 10 pack·years of cigarettes. The patients with asthma and HC were nonsmokers. A summary of the subject demographics is presented in Table 1.

**Table 1.** Subject/patients demography and lung function data

	Group					
Parameter	HC	ACO	Asthma	COPD -CS	COPD -ES	NLFS
Patients/subjects, *n*	28	12	14	11	12	13
GINA diagnosis: mild persistent/moderate/severe, *n*	N/A	0/0/4	5/1/8	N/A	N/A	N/A
ICS treatment, *n*	0	7	6	0	0	0
ICS dose, mcg/day	0	800	750	0	0	0
No. of OCS courses, *n*	0	1.5 (0–10)	2 (0–6)	0	0	0
Gold diagnosis: Stage I and II/Stage III, *n*	N/A	7/1	N/A	11/0	12/0	N/A
Sex (M/F), *n*	9/20	6/6	6/8	7/4	6/6	9/4
Age, yr	61 (20–76)	70 (52–77)	62 (26–81)	60 (52–69)	62 (53–69)	46 (30–66)
Smoking history, pack·years	0	22.5 (15–103)	0	46.3 (18–78.8)	54.8 (18.0–150.5)	35 (10.5–57)
FEV_1_ %predicted	99.5 (75–129)	58 (35–96)	81.5 (48–108)	82.7 (68–100)	80.0 (54.5–104.7)	98 (78–114)
FEV_1_/FVC%	78.4 (67–88)	65.5 (31–84)	74 (52–90.2)	63.9 (46.6–67.6)	58.5 (38.0–68.0)	76 (69–96)

Values are median and range. ACO, asthma COPD overlap; COPD, chronic obstructive pulmonary disease; COPD-CS, COPD current smokers; COPD-ES, COPD exsmokers; FEV_1_, forced expiratory volume in 1 s; FVC, forced vital capacity; GINA, Global Initiative for Asthma; HC, healthy control; ICS, inhaled corticosteroids; *n*, no. of subjects/patients; N/A, not applicable; NLFS, normal lung function smokers; OCS, oral corticosteroids.

### Histochemical Staining

At first, formalin-fixed, paraffin-embedded blocks were sectioned at 3 µm. Then, sections were dried overnight, deparaffinized, and subsequently stained with Masson’s trichome. The staining protocol included treatment with Bouin fluid at 56°C for an hour and washing for 2 min under tap water. This was followed by Weigert’s nuclear staining iron hematoxylin treatment for a further 8 min. Following this, tissues were washed with deionized water and then treated with Biebrich scarlet-acid fuchsin for 3 min to stain muscle and collagen. Next, the tissues were washed with deionized water for 3 min, differentiated in the phosphomolybdic-phosphotungstic acid solution for 3 min, and subsequently treated in aniline blue for 3 min to stain collagen. Later, the tissues were dedifferentiated in 1% acetic acid for 2 min, followed by dehydration. All stained slides were randomly coded by a person independent of the study.

### Quantification of Stained Tissue

Computer-assisted image analysis was performed with a Leica DM 500 microscope (Leica Microsystems, Germany), Leica ICC50W camera, and Image-Pro Plus 7.0 software, generally adhering to the American Thoracic Society and European Respiratory Society (ATS/ERS) standards for quantitative assessment of lung structure (20). Stained tissues with visible epithelium, RBM, and LP area were selected for image analysis. At first, two-dimensional images of entire regions of the stained tissue were captured at ×40 and ×10 magnifications. After that, five random images (×40) per subject were selected for cell counts, RBM, and epithelial thickness assessments. Finally, ×10 images were used for their area measurement for smooth muscles. The observer was blinded to the subject and diagnosis. We have previously published research articles using these methods (12, 21–23).

#### Thickness measurements.

**Figure 1.**
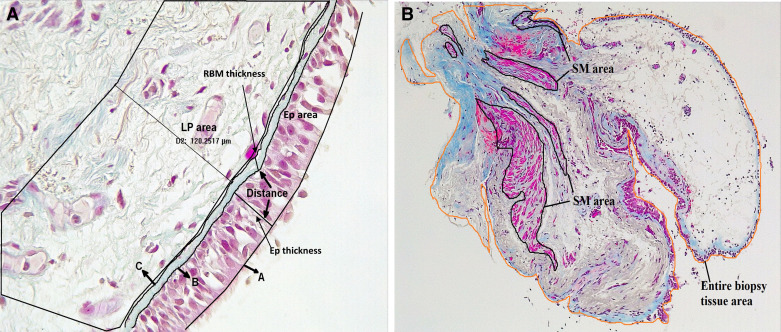
*A*: representative image (×40 magnification) of large airway describing the epithelial (EP) area, lamina propria (LP) area (120-µm deep), and distance between two trace lines. In case of epithelial and reticular basement membrane (RBM) thickness, three trace lines: one at apical (A) and another at the basal surfaces (B) of epithelium, and another trace line at outer limit of lamina reticula (C) were drawn using automated software Image-Pro Plus. The average distance (in µm) between two lines was measured. *B*: representative image (×10 magnification) of large airway tissue describing the smooth muscle (SM) areas and entire biopsy tissue area.

Epithelial and RBM thickness measurements were conducted based on the orientation of the tissue. First, three trace lines were drawn at the epithelium’s apical and basal surfaces, and the reticular lamina’s outer limit. Next, using the automated program of tissue image analysis software Image-Pro Plus v7, the average distance between the trace lines at apical and basal surfaces was calculated for epithelial layer thickness. Similarly, the average distance between the trace lines at the basal epithelial surface and the lower limit of the reticular lamina was calculated for RBM thickness. Details are provided in Fig. 1.

#### Cell counts.

Goblet cells, total epithelial cell count, total cell count within the RBM, and total cells in lamina propria (LP) up to 120-µm deep into the tissue were enumerated (Fig. 1*A*). The epithelial, goblet, and RBM cells were presented per mm of RBM length. For total cell counts in the LP and RBM, hematoxylin‐stained sizeable nuclei were considered individual cells, excluding microcapillaries and vessel cells. In the LP, cells were counted from a polygonal area selected using the Image-Pro Plus software and presented per mm^2^.

#### Smooth muscle area.

Using the polygon area selection tool of Image-Pro Plus software, outlines surrounding all the visible smooth muscles were drawn, and the software reported area values were considered for the total smooth muscle area in the image. Similarly, the area for the entire biopsy tissue was measured. Finally, the average smooth muscle area was adjusted for the whole biopsy tissue area and presented as the ratio (Fig. 1*B*).

### Statistical Analysis

Following a normal distribution check (D’Agostino and Pearson test), a nonparametric (Kruskal–Wallis) analysis of variance (ANOVA) with multiple comparisons using Dunn’s test was used to check the intra- and intergroup variance. In addition, a nonparametric Mann–Whitney test was performed to check the individual group differences compared with ACO. Results are reported as median and range unless otherwise mentioned. Based on the ANOVA result, univariate Spearman *r* was used for correlation analysis. A *P* value of <0.05 was considered significant. All analyses were done using GraphPad Prism v9 (San Diego, CA).

## RESULTS

**Figure 2.**
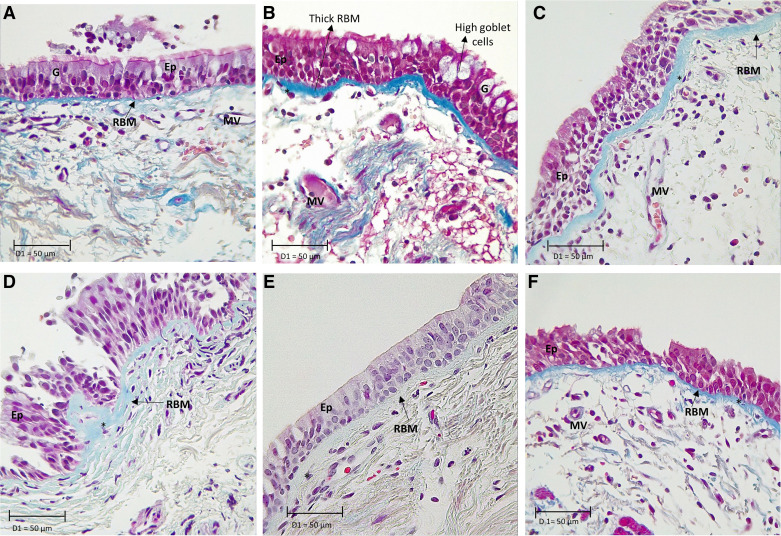
*Top*, *left* to *right*: Masson trichrome-stained sections of large airway tissue from healthy control (*A*), asthma-COPD overlap (*B*), and asthma (*C*). *Bottom*, *left* to *right*: COPD current smokers (COPD-CS) (*D*), COPD exsmokers (COPD-ES) (*E*), and normal lung function smokers (NLFS) (*F*). Images are representative of tissues obtained from healthy control, NLFS, and pathological groups in ×40 magnification. Nuclei and muscle: magenta; RBM and collagen: blue; RBC: orange. Note thickened RBM, increased goblet cells, hypocellularity in asthma-COPD overlap airways. COPD, chronic obstructive pulmonary disease; Ep, epithelium; G, goblet cells; RBM, reticular basement membrane; MV, microvessels. *RBM cell. Scale bar, 50 µm.

We characterized remodeling changes in all the three layers of the large airway mucosa from patients with ACO. We compared HC, asthma, COPD-CS and ES, and smokers with normal lung function (Table 2). Individual assessments for the epithelium, RBM, and LP are detailed here. The representative tissue micrographs are provided in Fig. 2, *A–F*.

**Table 2.** Findings of airways remodeling parameters in healthy, ACO, asthma, COPD-ES, COPD-CS, and NLFS at a glance

Morphometric Parameter	HC	ACO	Asthma	COPD-ES	COPD-CS	NLFS
Epithelial thickness	+	++	+	+	+++	+++
Epithelial cells	+	+	+	+	++	+++
Goblet cells	+	+++	++	+	+	+
RBM thickness	+	++	+	+	+++	++
RBM cells	+	++	+++	+	+	+
LP cellularity	+	++	+++	++	+	+
SM area	+	+	+++	+	++	+++

ACO, asthma COPD overlap; COPD, chronic obstructive pulmonary disease; CS, current smokers; ES, exsmokers; HC, healthy control; LP, lamina propria; NLFS, normal lung function smokers; RBM, reticular basement membrane; SM, smooth muscle. Each + represents the degree of presence of morphometric parameters with disease: “+” means less severe, “++” is moderately severe, and “+++” is very severe.

### Changes to the Epithelial Layer

#### Epithelial thickness.

**Figure 3.**
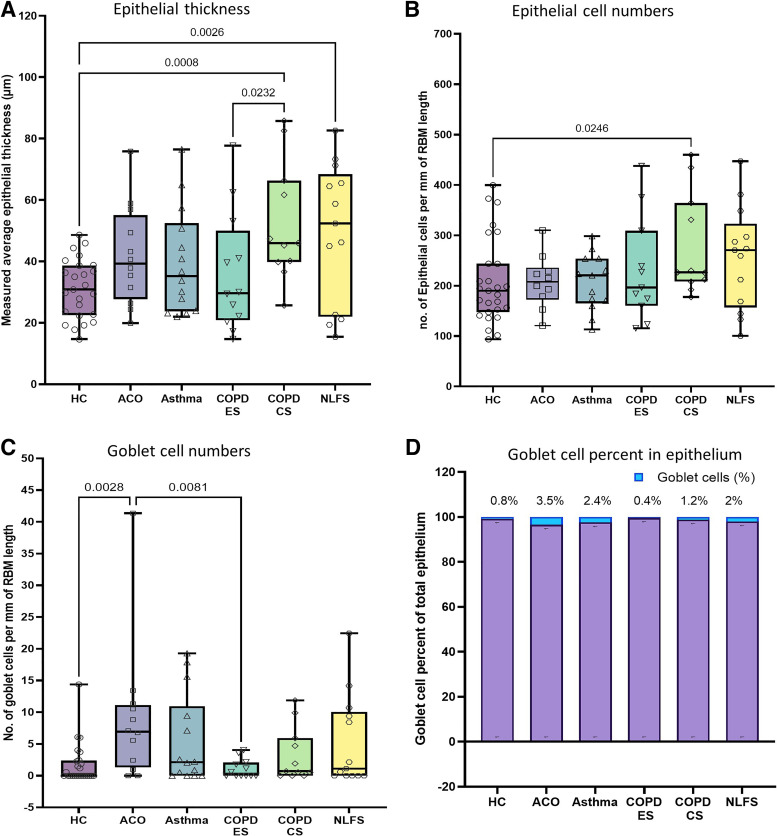
Box plots showing the ANOVA results for epithelial thickness (*A*), epithelial cells/mm reticular basement membrane (RBM) (*B*), and goblet cells per mm of RBM (*C*) in healthy control (HC), patients with asthma COPD overlap (ACO), asthma, COPD exsmokers (COPD-ES), COPD current smokers (COPD-CS), and normal lung function smokers (NLFS). The box-and-whisker plots showing the minimum and the maximum value, the lower and the upper quartile and the horizontal line as median. *P* < 0.05 is significantly different. Insignificant *P* values are not shown in the plot. Column plot (*D*) showing percent contribution of goblet cells in the epithelial cell population. COPD, chronic obstructive pulmonary disease.

In our intergroup analysis (ANOVA), we noted a thicker epithelium (median [range]) in the ACO group (39.3 [19.9–75.9] µm) than in HC (30.8 [14.7–48.6] µm), asthma (35.3 [20.0–76.5] µm), and COPD-ES (29.7 [14.7–77.7] µm). ACO epithelium was, however, thinner than both COPD-CS (46.0 [25.7–85.8] µm) and NLFS (52.4 [15.4–82.6] µm), although none were statistically significant (Fig. 3*A*). On applying the Mann–Whitney one-to-one comparison test, significance in thickness was seen in ACO compared with HC (*P* = 0.0263), but not with other groups (Table 3).

**Table 3.** Individual group differences: comparison to ACO using Mann–Whitney U test

Morphometric Parameter	Group	Median Difference (95% CI)	*P* Value
Epithelial thickness	HC	8.42 (−0.31 to 19.61)	0.0263*
	Asthma	−3.995 (−15.58 to 10.32)	0.3523
	COPD-ES	−9.55 (−20.60 to 8.08)	0.1737
	COPD-CS	6.71 (−4.14 to 25.67)	0.0847
	NLFS	8.86 (−11.38 to 27.23)	0.2186
Epithelial cells	HC	17.49 (−45.41 to 59.14)	0.2665
	Asthma	13.13 (−52.92 to 49.78)	0.4879
	COPD-ES	−11.32 (−61.96 to 93.92)	0.4863
	COPD-CS	19.01 (−7.57 to 149.9)	0.0661
	NLFS	63.24 (−36.22 to 121.1)	0.1284
Goblet cells	HC	6.77 (0.95 to 8.80)	0.0010*
	Asthma	−4.65 (−6.77 to 3.85)	0.2803
	COPD-ES	−5.58 (−8.83 to −0.66)	0.0043*
	COPD-CS	−6.06 (−7.1 to 0.55)	0.0843
	NLFS	−5.56 (−6.77 to 2.82)	0.2439
RBM thickness	HC	5.22 (2.63 to 7.01)	<0.0001*
	Asthma	−1.69 (−3.30 to 1.14)	0.2540
	COPD-ES	−1.46 (−3.96 to 1.41)	0.2179
	COPD-CS	3.74 (−1.18 to 12.72)	0.0759
	NLFS	−2.80 (−4.42 to 0.11)	0.0252*
RBM cells	HC	20.59 (10.49 to 23.98)	0.0002*
	Asthma	3.41 (−6.73 to 12.08)	0.2334
	COPD-ES	−13.37 (−20.62 to −3.24)	0.0018*
	COPD-CS	−14.03 (−21.65 to −5.41)	0.0014*
	NLFS	−19.66 (−26.24 to −12.38)	<0.0001*
Total LP cells	HC	506.2 (−426.7 to 915.5)	0.2256
	Asthma	1,427 (−317.5 to 2,573)	0.0613
	COPD-ES	−91.90 (−801.0 to 1,068)	0.4214
	COPD-CS	−898.8 (−1,551 to 37.38)	0.0396*
	NLFS	−725.5 (−1,315 to 288.5)	0.1473
Ratio of SM area to total tissue area	HC	−0.046 (−0.065 to 0.007)	0.0576
	Asthma	0.073 (0.037 to 0.215)	0.0008*
	COPD-ES	0.044 (−0.028 to 0.067)	0.1405
	COPD-CS	0.071 (0.022 to 0.107)	0.0035*
	NLFS	0.142 (0.019 to 0.192)	0.0030*

* Significantly different. ACO, asthma COPD overlap; CI, confidence interval; ES, exsmoker; COPD, chronic obstructive pulmonary disease; CS, current smokers; HC, healthy control; LP, lamina propria; NLFS, normal lung function smokers; RBM, reticular basement membrane; SM, smooth muscle.

Among the pathological groups, only COPD-CS (*P* = 0.0008) and NLFS (*P* = 0.0026) had significantly thicker epithelium than HC (Fig. 3*A*). We also noted a reduced thickening of epithelium in COPD-ES (*P* = 0.0232) than in COPD-CS.

#### Epithelial cells.

Similar to thickness findings, epithelial cell numbers in ACO (207.6 [120.8–310.1]) tended to be higher than in HC (190.1 [93.63–399.6]), COPD-ES (196.3 [115.5–437.6]), but contrastingly lower in asthma (220.7 [113.2–298.6]), though no statistical significance was observed in our intergroup analysis (ANOVA). ACO groups also had lower cell numbers than COPD-CS (226.6 [177.5–459.9]) and NLFS (270.8 [100.2–447.2]) (Fig. 3*B*). Furthermore, significantly higher epithelial cells were noted in COPD-CS (*P* = 0.0264) and NLFS (*P* = 0.0961) than in HC.

We found no significant differences with the ACO group in the one-on-one Mann–Whitney comparison test with other groups (Table 3).

#### Goblet cells.

We identified an increase in goblet cell numbers in ACO (6.77 [0–13.42]) that was significantly higher than in HC (0 [0–6.08], *P* = 0.0028) and COPD-ES (0.295 [0–4.05], *P* = 0.0081) in the intergroup analysis (ANOVA) (Fig. 3*C*). A significant increase in ACO over COPD-CS (0.71 [0–11.88], *P* = 0.0043) was also noted when analyzed using the Mann–Whitney test (Table 3). Furthermore, we demonstrate an increase in percent goblet cells in ACO, which constituted 3.5% of their total epithelial cell population compared with 2.4%, 1.2%, and 0.4% in asthma, COPD-CS and COPD-ES, respectively (Fig. 3*D*).

### Changes to the Reticular Basement Membrane

#### Reticular basement membrane thickness.

**Figure 4.**
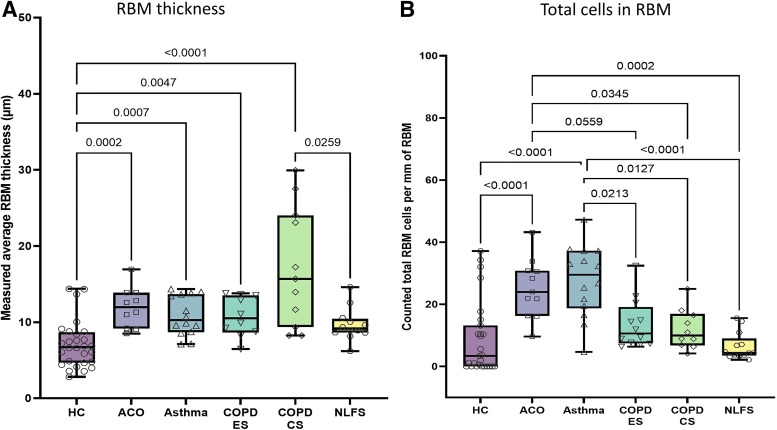
Box plots showing the ANOVA results for reticular basement membrane (RBM) thickness (*A*) and RBM cells per mm of RBM (*B*) in healthy control (HC), patients with asthma COPD overlap (ACO), asthma, COPD exsmokers (COPD-ES), COPD current smokers (COPD-CS), and normal lung function smokers (NLFS). The box-and-whisker plots showing the minimum and the maximum value, the lower and the upper quartile and the horizontal line as median. *P* < 0.05 is significantly different. Insignificant *P* values are not shown in the plot. COPD, chronic obstructive pulmonary disease.

Our intergroup analysis (ANOVA) showed that the ACO reticular basement membrane (RBM; 11.95 [8.5–16.95] µm) was significantly thicker than HC (6.74 [2.81–14.37] µm, *P* = 0.0002). Patients with ACO also tended to have a thicker RBM than patients with asthma (10.27 [7.12–14.34] µm), COPD-ES (10.49 [6.45–143.78] µm), and NLFS (9.15 [6.2–14.6] µm) but was thinner than patients with COPD-CS (15.69 [8.24–29.94] µm) group (Fig. 4*A*). Overall significant RBM thickness was observed in patients with asthma (*P* = 0.0007), COPD-CS (*P* < 0.0001), and COPD-ES (*P* = 0.0047) compared with HC, the only exception being NLFS (Fig. 4*A*). In addition, we noted significant RBM thickness in ACO compared with NLFS (*P* = 0.0252) when using the Mann–Whitney test (Table 3).

#### RBM cells.

We observed significant increase in cellularity in the ACO RBM (23.98 [9.57–43.19]) compared with HC (3.39 [0–37.16], *P* < 0.0001), COPD-ES (10.62 [6.33–32.40], *P* = 0.0559), COPD-CS (9.95 [4.14–24.92], *P* = 0.0345), and NLFS (4.32 [2.14–15.55], *P* = 0.0002) with ANOVA; however, their cell numbers were slightly lower in patients with asthma (29.52 [4.63–47.24]) (Fig. 4*B*). Among the pathologies, RBM cellularity in patients with asthma was the greatest wherein significant increases over COPD-ES (*P* = 0.0213), COPD-CS (*P* = 0.0127), NLFS (*P* < 0.0001), and HC (*P* < 0.0001) was noted (Fig. 4*B*). Similar significant changes were noted in one-to-one Mann–Whitney comparison between ACO and other groups that is represented in Table 3.

### Changes in Lamina Propria and Smooth Muscle Area

#### Lamina propria total cells.

**Figure 5.**
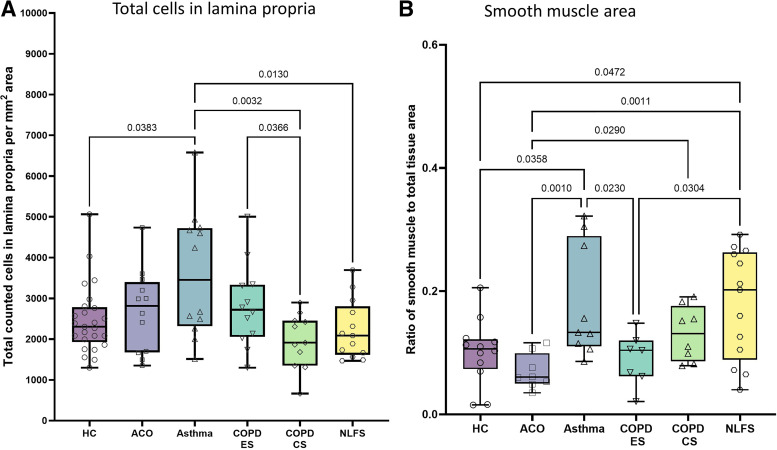
Box plots showing the ANOVA results for lamina propria (LP) total cells/mm^2^ (*A*) and smooth muscle area (*B*) in healthy control (HC), patients with asthma COPD overlap (ACO), asthma, COPD exsmokers (COPD-ES), COPD current smokers (COPD-CS), and normal lung function smokers (NLFS). The box-and-whisker plots showing the minimum and the maximum value, the lower and the upper quartile and the horizontal line as median. *P* < 0.05 is significantly different. Insignificant *P* values are not shown in the plot. COPD, chronic obstructive pulmonary disease.

Like RBM cell numbers, the lamina propria (LP) cells (per mm^2^) in patients with asthma were the greatest among the pathological groups, whereas cellularity in COPD-CS and NLFS groups was the lowest. The LP cell numbers in ACO (2,812 [1,351–4,738]) were higher than in HC (2,306 [1,302–5,062]), COPD-CS (1,913 [664.9–2,894]), NLFS (2,086 [1,468–3,694]), and COPD-ES (2,720 [1,300–5,003]), but lower than in asthma (4,239 [1,513–9,809]), although all were statistically insignificant in the intergroup analysis (ANOVA) (Fig. 5*A*). However, the Mann–Whitney test did show a significant difference in ACO LP cell numbers than COPD-CS (*P* = 0.0396) (Table 3).

Asthma LP cell numbers were significantly higher than COPD-CS (*P* = 0.0032) and NLFS (*P* = 0.0130) LP numbers (Fig. 5*A*). Furthermore, a significant increase in LP cell number in COPD-ES compared with COPD-CS (*P* = 0.0366) was noted (Fig. 5*A*).

#### Smooth muscle area.

In the intergroup analysis (ANOVA), we noted a significantly reduced smooth muscle area in ACO (0.061 [0.035–0.116]) as compared with asthma (0.133 [0.086–0.322], *P* = 0.001), COPD-CS (0.131 [0.079–0.191], *P* = 0.0290), and NLFS (0.202 [0.04–0.292], *P* = 0.0011); however, the reduction was insignificant compared with HC (0.107 [0.015–0.206]) or COPD-ES (0.104 [0.021–0.148]) (Fig. 5*B*). Our one-on-one analysis using Mann–Whitney test resonates the intergroup analysis result with a significantly reduced smooth muscle in ACO than in asthma, COPD-CS, and NLFS (Table 3).

Furthermore, our intergroup analysis noted that the maximum smooth muscle area in asthma was significantly higher than in HC and COPD-ES (Fig. 5*B*).

#### Correlations.

We found a negative correlation between RBM thickness (Spearman *r* = −0.3000, *P* = 0.1857) and RBM cells (Spearman *r* = −0.2636, *P* = 0.2174) with FEV_1_/FVC, although the comparisons were not statistically significant. We also noted an insignificant negative correlation between goblet cells and FEV_1_/FVC (Spearman *r* = −0.1429, *P* = 0.3913).

#### Effect of inhaled corticosteroids treatment on morphometric changes in patients with asthma and ACO.

**Figure 6.**
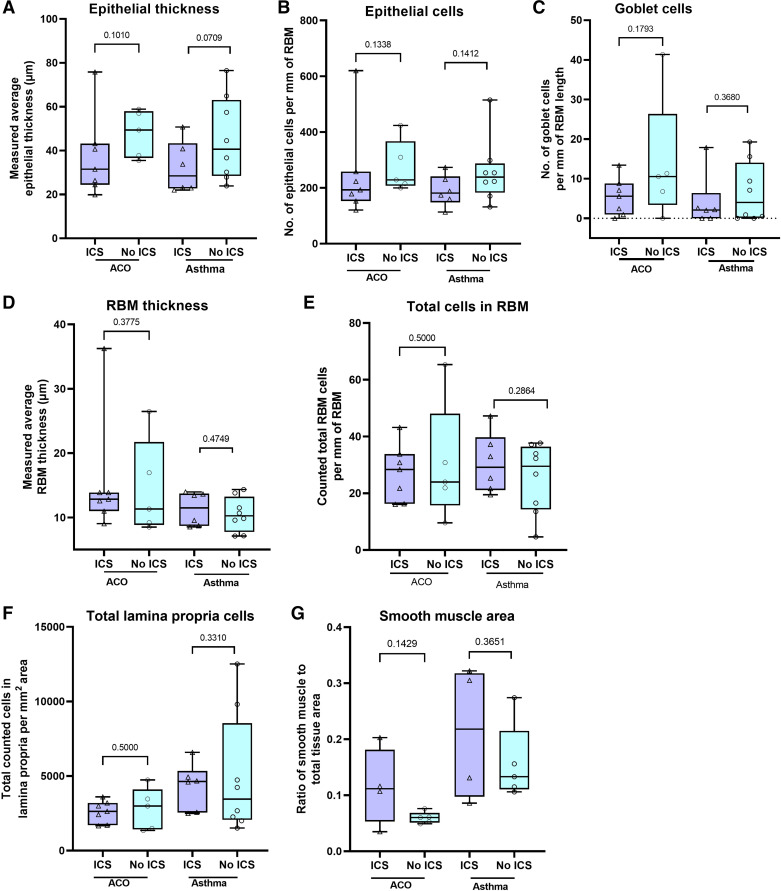
Box plots showing the Mann–Whitney test results for epithelial thickness (*A*), epithelial cells/mm reticular basement membrane (RBM) (*B*), goblet cells/mm of RBM (*C*), RBM thickness (*D*), RBM cells/mm RBM (*E*), lamina propria cells/mm^2^ (*F*), and smooth muscle area (*G*) in patients with asthma COPD overlap (ACO) and asthma with ICS and no-ICS. The box-and-whisker plots showing the minimum and the maximum value, the lower and the upper quartile and the horizontal line as median. *P* < 0.05 is significantly different between ICS and no-ICS groups. COPD, chronic obstructive pulmonary disease. ICS, inhaled corticosteroids.

As the patients with asthma and ACO were treated with inhaled corticosteroids in our study, we further evaluated if the treatment affected morphometric changes in them. Although the results were variable across the morphometric parameters, we did not find any significant difference between inhaled corticosteroids (ICS)-treated and -nontreated asthma and the ACO group using the Mann–Whitney test (Fig. 6, *A–G*).

## DISCUSSION

In the absence of universally accepted definition, the diagnosis of ACO generally becomes subjective involving the assessment of medical interview and conventional spirometry test (1, 2). However, these assessments are often inadequate to differentiate ACO from asthma and COPD. Therefore, identifying the distinctive airway remodeling patterns and cellular changes among clinical phenotypes of asthma, COPD, and ACO are essential to understand the underlying mechanisms and providing better patient management using appropriate interventions. This cross-sectional study is the first to quantitatively characterize the airway morphological changes using large airway EBB samples from patients with distinguishable phenotypes of chronic airways diseases: ACO, asthma, and COPD. Our study suggests a unique airway remodeling pattern in patients with ACO that encompasses features characteristically seen in either asthma or COPD (Table 2).

In our analysis of morphometric parameters in various layers in the large airway wall, we identified the most remarkable changes within the RBM. Our observation of thickened RBM in ACO agrees with previous EBB findings with ACO (19, 24). However, previous ACO findings lacked a comparison between smokers and healthy nonsmokers. Furthermore, CT studies (17, 18) also indicated a thicker airway wall in ACO than in either COPD or asthma. The RBM thickness data of our study revealed a dominant COPD-like phenotype in patients with ACO. Interestingly, RBM fragmentation occurs in COPD because of EMT, which warrants further investigations in ACO. Chronic inflammatory conditions in COPD and asthma could increase fibrogenic growth factors like TGF-β, EGF, and VEGF, all implicated in RBM thickness (13, 25, 26). Furthermore, the extracellular matrix components such as collagen I and III, tenascin, and fibronectin are instrumental in subepithelial fibrosis in asthma (25); however, in COPD, changes to the RBM composition in the large airway are less studied. Our previous study reported a thicker airway wall, increased αSMA-positive myofibroblast populations and their direct correlation between pathological changes in the ECM “scar” proteins, collagen I, and fibronectin (7). Though the usual pathognomonic of asthma is homogeneous thickened RBM (25, 27), we found significantly thickened RBM in COPD-CS than in HC, which contradicts Senhorini et al. (28), who reported a thinner large airway RBM in COPD than in fatal asthma and HC. A possible reason behind our observation was that patients with ACO and asthma were treated with corticosteroids, which could affect RBM thickness (29, 30).

The literature has reported convincing evidence on the roles of innate and adaptive inflammatory cells in asthma and COPD disease pathogenesis (8, 16). Our finding of higher RBM cells in patients with ACO and asthma could be a result of an active transepithelial egression of inflammatory cells through RBM (31). However, a possible role of EMT process could not be ruled out in the patients with ACO as our group has previously observed active EMT in patients with COPD (32, 33). Thus far, no previous study quantitatively generated total cellularity data in the ACO LP. Recently, an EBB study demonstrated a mild-to-moderate tissue lymphocyte and eosinophilic infiltration in ACO that were similar to asthma or COPD (19). We strongly believe that the nature of the cell type and their consequence on the RBM-related changes in ACO warrants further investigations. Our current findings of the total LP cells in HC, COPD, and NLFS agree with our previously reported data wherein we noted a marked decrease of LP cells in smokers and patients with mild-to-moderate COPD (8). The present finding of a high number of total LP cells in asthma could reflect the disease pathology, and to date, studies have shown the increased cellularity and abundance of inflammatory cells such as eosinophils and mast cells in the asthmatic airway (34–36).

The airway smooth muscle plays an indispensable role in airway remodeling, possibly by producing and modulating the ECM, controlling the cellular immune setting, cell proliferation, and hypertrophy (25). In both asthma and COPD, increased smooth muscle thickness has been reported as one of the characteristic features of airway remodeling (37, 38). Although a previous study (19) reported no difference in airway smooth muscle mass in the airways of patients with ACO, asthma, and COPD, our study, on the contrary, observed a significantly reduced smooth muscle area in the ACO group than in asthma, COPD-CS, and NLFS groups. The increase in smooth muscle thickness in asthma has been reported due to hypertrophy and hyperplasia of smooth muscle cells (39), but the evidence does not support hyperplasia of smooth muscle cells being the main cause of SM thickness in asthma (40). Although augmentation of smooth muscle was previously reported in the resected small airway and cartilaginous airways of patients with COPD (7, 41, 42), the study by Ebina et al. (43) reported mild hypertrophy in LA of patients with COPD. Our findings in patients with ACO require further investigations.

Mucus secretion under normal conditions has a protective action on the airway by moistening the air. However, the stress conditions such as infection, oxidative stress, smoking, and pathogenic factors, could lead to secretory cell hypertrophy and goblet cell hyperplasia due to prosecretory factors and confer mucous metaplasia (overproduction of mucin) (44, 45). We noted a significant increase in goblet cells in the patients with ACO compared with HC and COPD current and exsmokers. Our finding contrasted with those of Papakonstantinou et al. (19), who showed no differences between COPD with or without asthma features, but the authors reported significantly high goblet cells in COPD compared with asthma. Nonetheless, we have not found any significant difference between the patients with ACO and asthma or between the patients with COPD and asthma. Furthermore, the percentage contribution of goblet cells in the ACO epithelium, maximum among the group, could be an indication of increased mucous hypersecretion possibly due to stress condition or inflammatory response.

Several of our patients with asthma and ACO in the study were treated with ICS, and to check the modulation effect on airway remodeling, we have dichotomized the analysis of data from asthma and ACO groups based on ICS or no-ICS treatment. The investigation did not reveal any significant differences for the morphometric parameters between patients with ICS or no-ICS, which may be due to small sample size. Therefore, a comprehensive separate study is warranted to confirm the effect of ICS on airway remodeling.

The current study had limitations. We have not evaluated squamous metaplasia that is generally seen in smokers. Furthermore, this study has the limitation of type II error; however, we have observed significant differences representing robust distinctions between the groups. Above all, such invasive human clinical studies are challenging, especially with “rare tissue” from patients with ACO.

In conclusion, there is an urgent unmet need to characterize the ACO phenotype pathologically, especially compared with the contributing pathological groups of asthma and COPD. Our study is the first in-depth quantitative analysis of tissue morphology conducted on patients with ACO. The findings suggest that patients with ACO have differential airway remodeling changes that appeared to be severe. Furthermore, patients with ACO show distinct airway changes compared with patients with asthma and COPD. We believe that our work provides valuable histopathological evidence on the airway wall changes in patients with ACO, which will help clinicians in informed decision-making for diagnostic purposes and better patient management by reducing airway remodeling and subsequently the disease severity with appropriate therapeutic interventions.

## DATA AVAILABILITY

Data will be made available upon reasonable request.

## GRANTS

This work was supported by Clifford Craig Foundation Launceston General Hospital.

## DISCLOSURES

S. S. Sohal reports personal fees from Chiesi outside the submitted work. None of the other authors has any conflicts of interest, financial or otherwise, to disclose.

## AUTHOR CONTRIBUTIONS

S.S.S. conceived and designed research; S.D., W.L., S.J.B., A.V.G., and P.B. performed experiments; S.D., J.L., C.C., G.H., S.M., M.S.E., and S.S.S. analyzed data; S.D., W.L., P.A.B.W., M.S.E., and S.S.S. interpreted results of experiments; S.D. prepared figures; S.D. drafted manuscript; S.D., W.L., H.C.W., S.Y., P.S.P., P.A.B.W., M.S.E., and S.S.S. edited and revised manuscript; S.D., W.L., H.C.W., S.Y., J.L., C.C., G.H., A.V.G., P.B., P.S.P., P.A.B.W., M.S.E., and S.S.S. approved final version of manuscript.
